# Hepatic vena cava syndrome: a case report and literature review

**DOI:** 10.1093/omcr/omaf035

**Published:** 2025-05-28

**Authors:** Muhammad Zahid, Mohammad Es-Salim, Moaz O Moursi, Nabil Mahmood, Abdel-Naser Elzouki

**Affiliations:** Department of Internal Medicine, Hamad General Hospital, Doha, Qatar; College of Medicine, QU Health, Qatar University, Doha, Qatar; Weill Cornell Medical College, Qatar; Department of Internal Medicine, Hamad General Hospital, Doha, Qatar; Department of Internal Medicine, Hamad General Hospital, Doha, Qatar; College of Medicine, QU Health, Qatar University, Doha, Qatar; Weill Cornell Medical College, Qatar; Department of Radiology, Hamad General Hospital, Doha, Qatar; Department of Internal Medicine, Hamad General Hospital, Doha, Qatar; College of Medicine, QU Health, Qatar University, Doha, Qatar; Weill Cornell Medical College, Qatar

**Keywords:** hepatic venous outflow tract obstruction (HVOTO), hepatic vena cava syndrome (HVCS), membranous obstruction of the inferior vena cava, liver cirrhosis, case report

## Abstract

Background: Hepatic vena cava syndrome (HVCS) is an uncommon condition, with no prior reports from the Gulf area. In regions with limited resources, where this disease is found, there is a lack of data due to underdiagnosis. HVCS results from recurrent bacterial thrombophlebitis of the hepatic portion of the inferior vena cava (IVC), causing intimal thickening and obstruction, eventually leading to complications like liver cirrhosis and hepatocellular carcinoma.

Case presentation: A 32-year-old Afghan refugee lady, presented with progressive abdominal distention. Initial investigations showed anemia, splenomegaly, and massive ascites. Further investigations revealed cirrhotic liver, varices, and a stenotic segment of the IVC. She was diagnosed with HVCS, underwent IVC stenting, and commenced on clopidogrel and dabigatran.

Conclusion Often misdiagnosed, HVCS requires a high index of suspicion and should be considered in patients with liver cirrhosis with low socioeconomic backgrounds. Early diagnosis can prevent liver cirrhosis and hepatocellular carcinoma.

**Key clinical message**

Often misdiagnosed, Hepatic Vena Cava Syndrome requires a high index of suspicion and should be considered in low socioeconomic backgrounds. Early diagnosis can prevent liver cirrhosis and hepatocellular carcinoma.

## Introduction

Hepatic venous outflow tract obstruction (HVOTO) is an uncommon vascular disease that leads to a veno-centric form of liver cirrhosis. The three primary diseases of HVOTO are sinusoidal obstruction, Budd-Chiari syndrome, and hepatic vena cava syndrome (HVCS) [1]. Sinusoidal obstruction or veno-occlusive disease, a rare occurrence, is caused by pyrrolizidine alkaloid ingestion and is rarely observed in patients undergoing myeloablative therapy, chemotherapy, hematopoietic stem cell transplantation (HSCT), drug toxicity (e.g. 6-thioguanine), and recreational drug use (e.g. ‘poppers’) [2–4]. Budd-Chiari syndrome, more prevalent in Caucasian females, is caused by thrombosis of the hepatic vein and is associated with prothrombotic conditions [5].

HVCS, previously known as membranous obstruction of the inferior vena cava, is a chronic obstructive disease of the hepatic portion of the inferior vena cava (IVC). It results from recurrent bacterial thrombophlebitis that often begins in childhood. HVCS is more commonly reported in Afro-Asian countries among individuals living in poor hygienic conditions. It affects all age groups and both sexes. HVCS is a chronic disease with an insidious onset, characterized by recurrent acute exacerbations related to acute infections, and can lead to complications such as liver cirrhosis and hepatocellular carcinoma [1, 6, 7]. This underscores the need for early detection and proactive management. HVCS frequently coexists with other acute and chronic liver diseases. Therefore, patients with liver cirrhosis from low socioeconomic status populations in developing countries should be evaluated for this associated condition. Here, we present a case of a 34-year-old Afghan female who presented with massive ascites, was diagnosed with HVCS, and underwent IVC stenting as a treatment.

## Case report

A 32-year-old Afghan refugee female presented to the emergency room complaining of sudden worsening abdominal distention. Halfway through her pregnancy, which ended two months prior via caesarean section, she reported increasing abdominal distention to her gynecologist in Afghanistan. Ultrasound studies confirmed ascites and a viable pregnancy. Due to the provision of poor health care services there, she was not investigated for the cause of ascites but started on Furosemide 40 mg twice a day. She delivered a healthy baby by caesarian section two months before this presentation. No written previous medical record was available to us.

On presentation to our hospital, she described the distention as progressive, not relieved by medications, and associated with mild discomfort. She did not complain of abdominal pain, nausea, vomiting, diarrhea, or constipation. She also denied having fever, night sweats, orthopnea, lower limb swelling, shortness of breath, facial puffiness, or dysuria.

Her past medical history was not significant apart from on-and-off abdominal discomfort without a confirmed diagnosis. She was married with three children. Her family history was unremarkable. She was a non-smoker with no history of alcohol or drug use. She spent her entire life in Afghanistan and recently arrived in Qatar as a refugee one month before her presentation.

On examination, her pulse was 100 beats/minute, afebrile, respiratory rate 18 breaths/minute, blood pressure 105/86 mmHg, and oxygen saturation 98% on room air. She looked pale, with no jaundice, palmar erythema, spider nevi, or dilated veins. Her chest was clear with good bilateral air entry, normal heart sounds with no murmurs, and no lower limb or sacral edema. Her abdomen was remarkably distended, with multiple linear striae noticed. There was no tenderness upon palpation; it was dull on percussion with positive shifting dullness. Due to marked ascites, organomegaly was difficult to ascertain.

Her complete blood count showed a picture of mild iron deficiency anemia and reactive thrombocytosis. Her coagulation profile and kidney and liver function tests were within the reference range (Table 1). A complete non-invasive liver screen, including viral serologies and autoimmune profile, iron studies, ceruloplasmin, and alpha antitrypsin levels, was sent, which returned as negative (Table 2). She reported her BMI was never very high, and her glucose levels were not in the diabetic range.

**Table 1:** Initial basic blood investigations.

Lab Test	Result	Unit	Reference Range
WBC	8.7	*10^3/ul	4–10
HB	11.5	gm/dl	12–15 mg/dl
MCV	79.8	—	83–101
Platelets	538	*10^3/ul	150–400
PT	12.4	Seconds	9.7–11.8
INR	1.2	—	—
Creatinine	67	Umol/l	44–80
Urea	3.6	Mmol/l	2.5–7.8
Sodium	134	Mmol/l	133–146
Potassium	3.4	Mmol/l	3.5–5.3
Bicarbonate	30	Mmol/l	22–29
ALT	8	U/l	0–33
AST	14	U/l	0–32
ALP	93	U/l	35–104
GGT	18	U/l	6–42
Albumin	25	Gm/l	35–50
Total Protein	80	Gm/l	60–80
Total bilirubin	13	Umol/l	0–21
Lipase	19	U/l	13–60
CRP	34.7	mg/l	0–5
Ferritin	297	Ug/l	12–160
Iron	5	Umol/l	6–35
TIBC	38	Umol/l	45–80
Transferrin	1.5	Gm/l	2–3.6
Fe% saturation	13%	—	15–45

HB: hemoglobin, MCV: Mean corpuscular volume, WBC: White blood cell count, INR: International normalized ratio, PT: Prothrombin time, ALT: Alanine transaminase, AST: Aspartate transaminase, GGT: Gamma-glutamyl transferase, CR: Creatinine, CRP: C-Reactive Protein.

**Table 2:** Liver disease workup.

Test	Result w/Units	Normal Range
Ammonia level	43 umol/l	11–51
Hepatitis B Surface antibody	Non-Reactive	
Hepatitis C antibody	Non-Reactive	
Anti-Nuclear antibody (CTD screen)	Negative	
Antineutrophil cytoplasmic antibodies	Negative	
Anti-Mitochondrial antibody	Negative	
Anti-Smooth Muscle antibody	Negative	
Anti-Liver Kidney Microsomes	Negative	
Ceruloplasmin	32 mg/dl	16–45
Alpha1–antitrypsin	237.9 mg/dl	90.0–200.0
Ferritin	297.0 ug/l	12–160
Schistosoma antibody	Negative	
Anti B2 Glycoprotein IgG	Negative	
Anti B2 Glycoprotein IgM	Negative	

**Figure 1:**
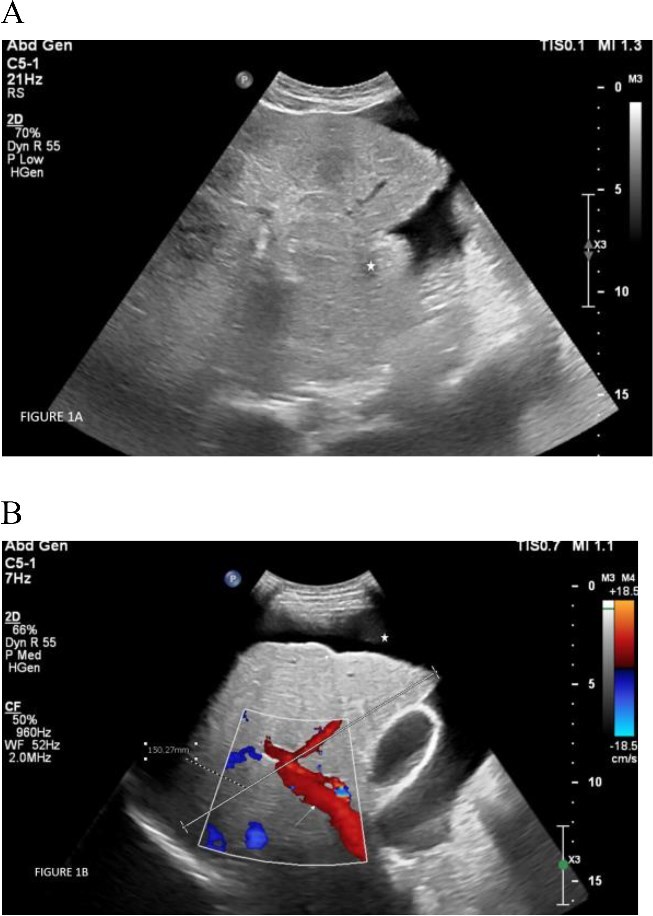
(A) Ultrasound examination of the liver demonstrating diffusely coarsened echotexture of the liver with an enlarged caudate lobe (white asterix). (B) Ultrasound study of the liver demonstrating diffusely coarsened echotexture of the liver with surface irregularity (short white arrow). The portal vein was patent (long white arrow). Massive ascites was demonstrated (white Asterix).

On ultrasonography (Fig. 1A and B), the liver measured 15 cm and demonstrated diffusely coarsened and heterogeneous echotexture, with surface irregularity and a prominent caudate lobe. The portal vein was patent and measured 11.3 mM. Splenomegaly (13.8 cm) was noted with massive ascites. Elastography assessment of the liver on ten samples by ElasPQ ultrasound shear wave showed a stiffness average of 16.26 kPa, indicative of moderate to severe liver fibrosis.

**Figure 2:**
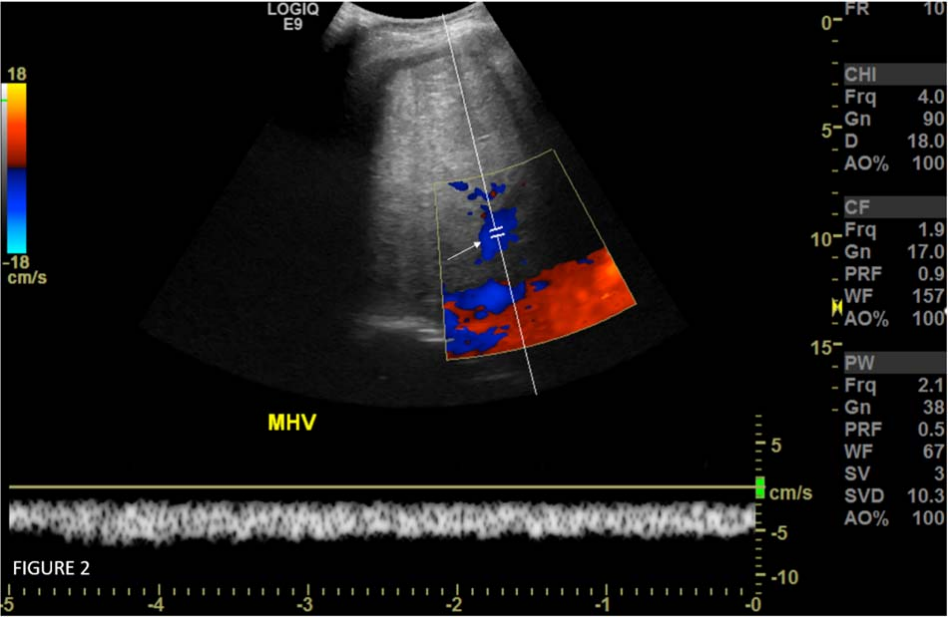
Spectral color doppler study of the middle hepatic vein (white arrow) prior to IVC dilatation shows normal flow direction towards the IVC but however abnormally dampened monophasic flow pattern.

On Doppler ultrasonography (Fig. 2), portal veins were patent and showed normal hepato-petal flow direction. Right, middle, and left hepatic veins were patent, showing a monophasic (HV2) type of minimal low-velocity flow with a Damping Index (DI) of 0.8. Normal hepatic arterial flow was seen.

Diagnostic and therapeutic abdominal paracentesis was performed. Ascitic fluid was yellow in color and slightly turbid, revealing an ascitic protein of 48.1 gm/l, ascitic albumin level of 12.2 gm/l, serum ascitic albumin gradient (SAAG) 12.8, LDH 118 U/l, and WBC 158/UL (5% neutrophils, 28% lymphocytes). The acid-fast bacilli smear and the tuberculosis PCR on ascitic fluid were negative. The facility to check for ascitic fluid adenosine deaminase was not available. However, ascitic fluid culture also returned as negative, including TB. A total of 32 liters of ascitic fluid was drained throughout her hospital stay.

Transthoracic echocardiography showed normal left ventricular function (59%), normal diastolic and valvular functions, and no regional wall motion defect or pericardial effusion. Right ventricle systolic pressure, right atrial pressure, and M mode—tricuspid annular plane systolic excursion (TAPSE) were 22.22 mmHg, 5 mmHg, and 19 mm, respectively.

**Figure 3:**
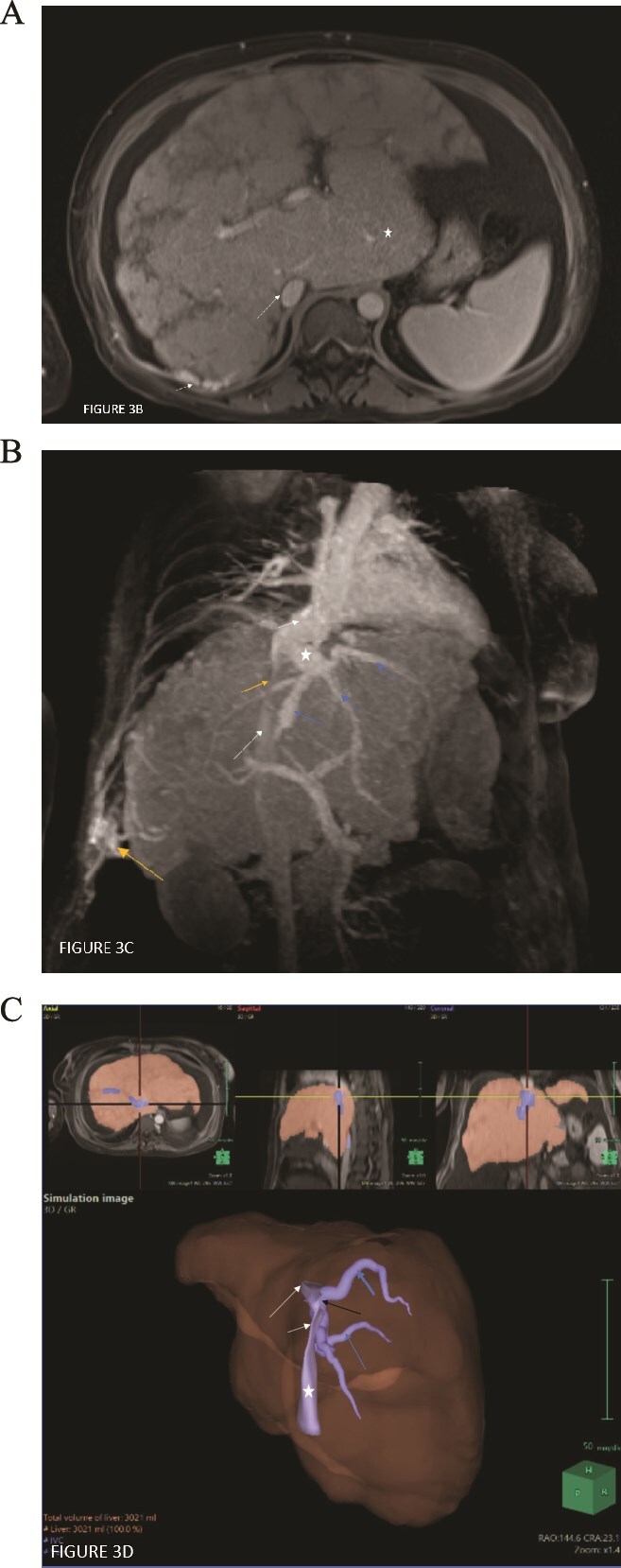
(A) Axial contrast T1W MRI study of the liver in the venous phase demonstrates an enlarged liver with surface lobulations and heterogenous reticular pattern of enhancement. A slit like severely narrowed intrahepatic segment of the IVC (long white arrow) is seen. The caudate lobe is enlarged (white star). Middle hepatic vein is denoted by the short white arrow. (B) Axial contrast T1W MRI study of the liver at a more inferior level in the venous phase demonstrates the enlarged caudate lobe (white star). The IVC is normal in caliber at this level (long white arrow) and is not compressed by the enlarged caudate lobe. Multiple venous collaterals are seen along the surface of the liver posteriorly (short white arrow). (C) Oblique MIP MRI image in the venous phase showing the hepatic veins (blue arrows) draining into a common confluence (white star) which subsequently drains into the supra-stenotic IVC (short white arrow), bypassing the stenotic segment of the intrahepatic IVC (short orange arrow). The IVC below the stenotic segment is depicted by the long white arrow and is normal in caliber. Venous collaterals are noted along the liver surface draining into the lower chest wall veins (long orange arrow). (D) Volume rendered MR image of the liver (rendered in brown), intrahepatic IVC (rendered in dark blue) and hepatic veins (rendered in light blue) demonstrates the short segment severe stenosis of the IVC (short white arrow) with normal caliber of the suprastenotic (long white arrow) and infrastenotic (white asterix) segments. The right (bold blue arrow) and middle (thin blue arrow hepatic veins appeared tortuous and were seen to fuse to form an alternate channel (black arrow) along with the left hepatic vein (not shown) that drained separately into the supra-stenotic segment of the IVC.

Magnetic resonance imaging (MRI) of the liver showed a cirrhotic liver with irregular contour and nodular surface, widening fissures, and marked architectural distortion. The liver had a heterogeneous mottled appearance and a reticulated architecture, with areas of hypo-enhancement in the arterial and portal venous phases that became relatively uniform in the delayed phase (Nutmeg liver). A stenotic segment involving the intrahepatic IVC was seen with venous collaterals. The hepatic veins appeared tortuous but patent, forming a confluence away from the stenotic segment of the IVC and draining superiorly into the supra-stenotic IVC segment. The inferior vena cava stenosis is very focal, and a grossly enlarged caudate lobe is seen at a more inferior level where the caliber of the IVC segment is normal. This indicates that the IVC segment stenosis is not caused by the enlarged caudate; rather, the caudate enlargement is a compensatory sequela of the IVC stenosis, which has caused cirrhosis and atrophy of the remainder of the segments (Figs 3A–D). The disease process of the intrahepatic IVC likely involved the segment, where the true anatomical confluence with hepatic veins initially was. This likely affected the hepatic venous outflow drainage, leading to cirrhosis and the development of collateral venous pathways within the liver parenchyma.

**Figure 4:**
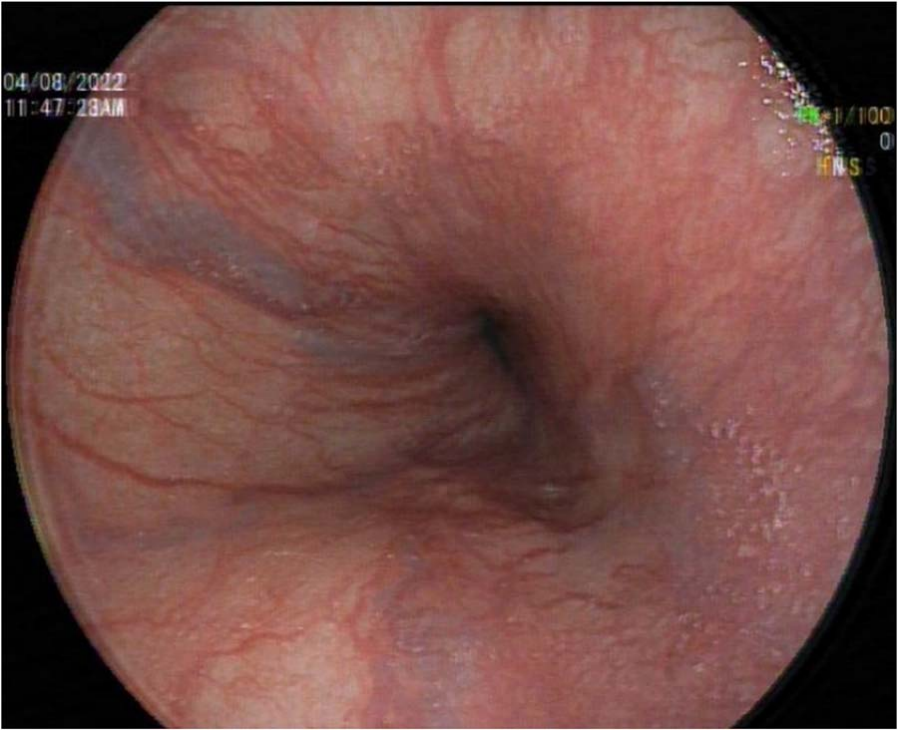
Endoscopic view of the lower Esophagus, showing 2 columns of grade 1 varices.

Upper Gastrointestinal tract endoscopy showed two columns of Grade 1 varices in the esophagus, normal gastroesophageal junction, and the stomach and duodenum look normal (Fig. 4).

**Figure 5:**
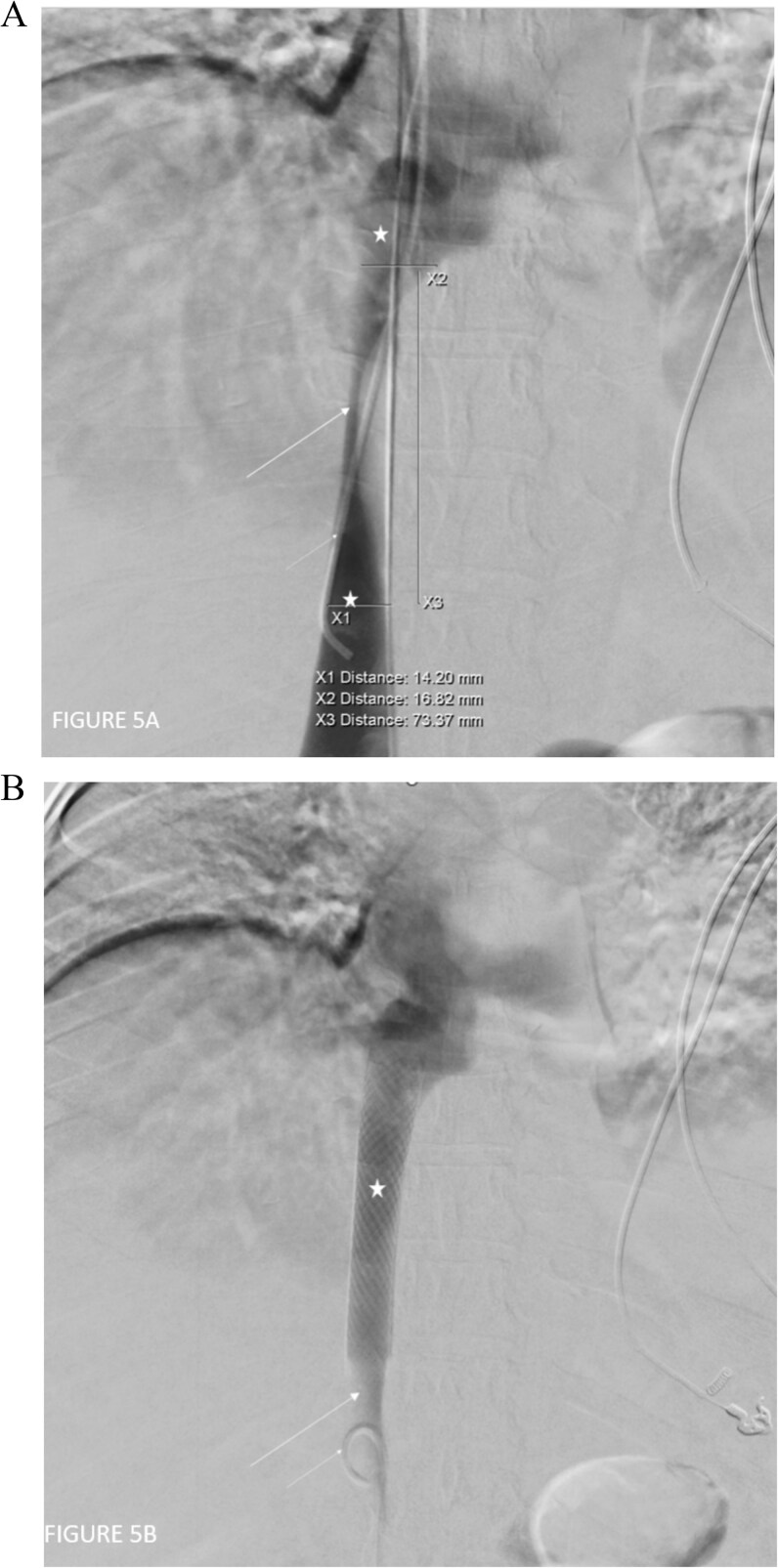
(A) Digital subtraction catheter IVC venogram study demonstrates the segment of severe narrowing in the intrahepatic IVC prior to stenting (long white arrow). There is contrast flowing across the stenotic segment with pre- and post-stenotic dilatation of the IVC (white star). The angiogram catheter can be seen straddling the stenotic segment with its tip in the infrastenotic segment (short white arrow). (B) Digital subtraction catheter IVC venogram study demonstrates the stent in situ within the previously stenotic segment (white star) which is now dilated to normal caliber. The tip of the catheter is noted in the infrastenotic segment (short white arrow) of the IVC. There is patent flow across the stent with resolution of the pre stenotic dilatation of the infrastenotic segment (long white arrow).

The angiogram shows stenosis of the intrahepatic IVC, with contrast flowing across the stenotic segment and pre and post-stenotic dilatation of the IVC, suggesting the stenosis is likely hemodynamically significant (Fig. 5A). The hepatic venous collateral pathway seen on MRI bypassing the stenotic segment and draining into the non-stenotic segment of the intrahepatic IVC are not visualized on the angiogram as there was no abnormal retrograde reflux into these collaterals from the non-stenotic segment of the IVC.

On day 3 of admission, the patient spiked a temperature of 39°C; blood and urine cultures were sent. The urine dipstick was negative for infection, and the chest x-ray was unremarkable. A Diagnostic ascitic fluid tap was repeated, which returned negative for spontaneous bacterial peritonitis. Blood cultures returned positive for methicillin-resistant *staphylococcus aureus* (MRSA) in 4 bottles, and intravenous vancomycin was initiated. A repeat transthoracic echocardiography was arranged, which was reported as normal, especially negative for any vegetation. She received intravenous vancomycin at a dose of 750 mg twice daily for a duration of two weeks as per hospital infectious disease guidelines, with regular monitoring of vancomycin levels. Following this treatment, subsequent blood cultures returned as negative. She remained stable and afebrile during the rest of her hospitalization.

**Figure 6:**
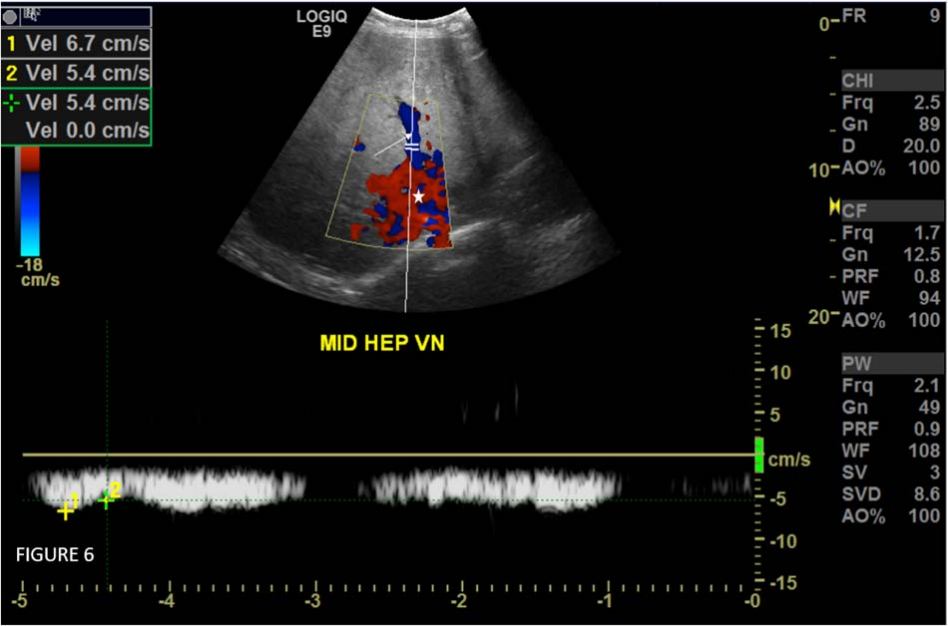
Spectral color doppler study of the middle hepatic vein (long white arrow) post IVC stenting shows normal flow direction towards the IVC (white asterix) with establishment of some, though blunted periodic flow pattern.

A multidisciplinary team consisting of internal medicine, hepatologist, hematologist, and interventional radiologist decided to go for venoplasty of the stenotic segment of IVC. Initially, an attempt to dilate the stenotic part of the inferior vena cava with a balloon failed; thereafter, a self-dilating stent was deployed (Fig. 5B). She was commenced on clopidogrel for six months and dabigatran, a direct oral anticoagulant, for life long. The post-procedure ultrasound Doppler scan confirmed the placement of the stent and its patency (Fig. 6).

We made the diagnosis of decompensated liver cirrhosis based on liver echotexture findings on imaging, splenomegaly, severe fibrosis on elastography, ascites, and grade 1 esophageal varices on upper gastrointestinal tract endoscopy. Her noninvasive liver screen returned negative, and imaging showed a stenotic segment of the intrahepatic inferior vena cava, which makes HVCS a likely underlying reason. She was started on spironolactone and furosemide for ascites and propranolol for primary prevention of esophageal varices.

At the 1-month follow-up appointment, she was doing well, her weight was stable, and she did not notice any noticeable abdominal distention during that period. The patient confirmed full compliance with the treatment and reported no side effects. Beyond this point, the patient was lost to follow-up as she left the country.

## Discussion

**Figure 7:**
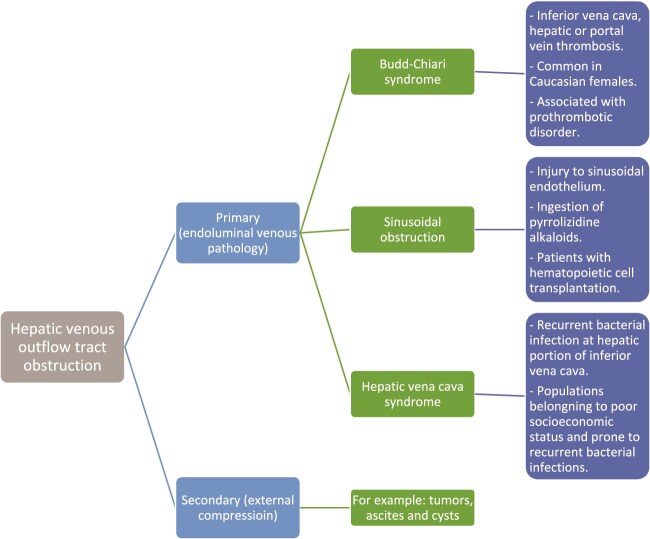
Types and common causes of hepatic venous outflow tract obstruction.

Hepatic microcirculation is a low outflow resistance system with a 49:1 pre-to-postcapillary resistance ratio [8]. Three large and nearly twenty small hepatic veins (HVs) drain the liver into the IVC, as HVs have no valves; any increase in IVC pressure leads to back pressure and liver injury [9]. HVOTO, as illustrated in Fig. 7, can occur at the level of sinusoids (sinusoidal obstruction), in HV (Bud Chiari syndrome), or in IVC at the HV outlets (Hepatic Vena Cava Syndrome) [9].

HVCS is a chronic condition mainly reported in populations living in poor hygienic conditions in Asia and Africa. It is caused by recurrent localized infective phlebitis of the hepatic portion of IVC, which, on resolution, transforms into stenosis or thick obstruction, leading to increased pressure in HVs and associated liver injury. Recurrent episodes of thrombotic phlebitis and hepatocyte injury ultimately lead to liver cirrhosis and can be further complicated by hepatocellular carcinoma [10].

The prevalence of HVCS in different countries is inversely proportional to the standard of hygiene [11]. In Japan, the incidence of HVCS was 8.1% in 1921, which dropped to 0.1% in 1986–87, likely secondary to better hygiene and healthcare standards [12, 13]. This condition is endemic in Nepal and is one of the most common causes of ascites and liver cirrhosis there.

Our patient was from Afghanistan, a war-affected country with a deficient healthcare system. According to the United Nations, about two-thirds of Afghanistan has a critical situation of water scarcity and poor hygiene status and is at high risk of further deterioration in hygiene and sanitation conditions [14]. Our patient presented with worsening ascites, and the workup revealed stenosis in the hepatic portion of IVC on imaging and severe liver fibrosis on elastography, while the rest of the noninvasive liver screen results were negative. HVCS should be considered as a potential diagnosis in all patients with liver cirrhosis from impoverished socioeconomic backgrounds. HVCS may be comorbid with other conditions like chronic viral hepatitis or alcoholism in patients with liver cirrhosis.

IVC is formed by the fusion of multiple branches of three different embryonic veins and is subject to developmental anomalies like duplication, remnants of embryonic tissue like webs and membranes, and abnormal connections, leading to the presumption that HVCS is a congenital disease [15].

Recurrent thrombophlebitis as the underlying pathogenesis of HVCS was determined from clinical and cartographic studies of patients with this disease and autopsy studies of historical cases. The histological evidence of thrombophlebitis of IVC and bacterial growth from thrombus in the IVC were reported. Pleasants of John Hopkins University (1911), Nishikawa et al. (1918), and Ridgon (1933), based on autopsy studies, suggested that the thrombophlebitis was the initial lesion which subsequently got organized, leading to shrinkage and stenosis of the involved IVC segment [15–18].

Gut infection and gram-negative bacteraemia are common in children with poor nutrition, pregnant ladies with pelvic infections, and poorly controlled diabetics. Bacteraemia leads to localized thrombophlebitis of a hepatic portion of IVC, a site more vulnerable to microscopic endothelial damage due to constant movement of the diaphragm and the turbulence in the blood caused by HV inflow perpendicular to the IVC at this segment. Damaged intima is at risk of further infection and thrombus formation [19].

Caudate lobe enlargement is reported as a cause of hepatic vein thrombosis, leading to the narrowing of a long segment of the hepatic portion of IVC [20]. Such lesions in patients at risk of recurrent bacteraemia were more likely to be stenosis secondary to thrombophlebitis, as reported by Takaishi et al. [21]. Recurrent thrombophlebitis leading to stenosis of a hepatic portion of vena cava is underdiagnosed due to greater emphasis on membranes in the IVC [22]. Etiology, disease progression, and HVCS management are different from classical BCS. Hence, it is identified as a separate cause of HVOTO after BCS and sinusoidal obstruction syndrome [23].

Past autopsy studies and the histology of the lesions in the IVC indicated the presence of bacteria within the thrombus in the IVC, along with features of thrombophlebitis. This supports the hypothesis that HVCS is initiated by a bacterial infection causing localized thrombophlebitis in the IVC, typically at the site of hepatic vein opening. Subsequent recurrent infections lead to stenosis and occlusion of hepatic veins and/or hepatic portion of the IVC [22, 24]. Ischemic necrosis and obliteration of hepatic parenchyma, secondary to hepatic vein and sinusoidal obstruction due to the organization of recurrent thrombophlebitis, ultimately leading to liver cirrhosis in HVCS [10].

The acute stage of the disease is often prone to misdiagnosis, and the disease becomes chronic with long asymptomatic periods and recurrent acute infections. Acute exacerbations are typically precipitated by bacterial infections, manifesting as upper abdominal pain, fever, mild jaundice, and elevated transaminases. Severe exacerbations present with fever, jaundice, ascites, peripheral edema, and variceal bleeding [25–27].

Our patient was presented with worsening ascites, a common presentation of acute exacerbation of HVCS precipitated by infection. The patient had a documented fever during her hospital stay, with positive blood cultures, treated promptly with antibiotics. Literature review reveals that ascitic fluid in acute exacerbation of HVCS has high protein content, high SAAG and often evidence of bacterial peritonitis [28]. Our patient had a diagnostic tap twice during the admission; the SAAG was 12.8 and 13, respectively, and was negative for any evidence of bacterial peritonitis on both occasions.

The diagnosis of HVCS is established through the identification of stenosis or complete occlusion of a hepatic portion of IVC by imaging. Ultrasonography and color Doppler examination of the liver and IVC are specific and sensitive to diagnose this condition, which is further confirmed by an inferior vena cavogram or MRI scan [1]. Depending upon the stage of the disease, liver biopsy findings can range from minimal findings to central vein dilatation or fibrosis, sub-lobular vein endophlebitis or thrombosis, or ultimately liver cirrhosis [29, 30]. Our patient had a negative non-invasive liver screen for chronic liver disease, and an MRI liver scan showed a 3.5 cm stenotic segment involving the intrahepatic IVC, thus strongly favoring the diagnosis of HVCS.

Acute exacerbation of HVCS is usually caused by acute infection and is treated with a prolonged course (4–6 weeks) of broad-spectrum antibiotics. Ascites is managed with spironolactone and furosemide and dietary restriction of salt. The use of prophylactic antibiotics to prevent acute exacerbation is not recommended. Recurrence can be prevented by adopting good hygiene, abstaining from alcohol, and maintaining good nutrition [31].

Previously, endovascular procedures as a treatment for HVCS were proven effective and safer. Nowadays, percutaneous transluminal angioplasty and stenting are performed for stenotic vascular portions [7]. Stenting is not uncommonly complicated by hepatic vein obstruction or stent thrombosis leading to acute worsening of ascites [32, 33]. In our case, a stent was deployed in the stenotic portion of the IVC, and anticoagulation therapy was initiated. At the 1-month follow-up, she was doing fine with well-controlled ascites.

## Conclusion

HVCS is one of the causes of HVOTO, caused by recurrent thrombophlebitis of the hepatic portion of the IVC, which, on resolution, leads to thickening of the intima and subsequent obstruction. This condition is commonly misdiagnosed, especially in the initial stages, necessitating a high suspicion index. Acute bacterial infection can precipitate acute exacerbations, presenting commonly with worsening jaundice, fever, and ascites. Diagnosis is confirmed with imaging showing obstruction of the hepatic portion of the inferior vena cava. Treatment involves prolonged antibiotics for acute bacterial exacerbations, diuretics, salt restriction for ascites, angioplasty, and stenting for stenotic vascular segments, in addition to anticoagulation in some patients. Notably, HVCS can be a comorbid condition in cirrhotic patients from other etiologies, making it important to be considered in the differential diagnosis, especially in patients from developing countries with low socioeconomic status presenting with ascites, with or without liver cirrhosis. Early diagnosis of this disease can prevent progression to liver cirrhosis and hepatocellular carcinoma. Further research is needed to improve our understanding, enhance diagnosis and treatment, and eventually achieve better patient outcomes.

## Data Availability

Data sharing is not applicable to this article as no datasets were generated or analyzed during the current study.
